# Evaluation of Breast Lesions Using Multiparametric 3-Tesla Magnetic Resonance Imaging: A Cross-Sectional Study

**DOI:** 10.7759/cureus.108076

**Published:** 2026-04-30

**Authors:** Kavitha B, Thomson N Luwang, Maimom Tamphasana, Sarvesh R Ramanathan, Thendral G, Meenakumari Ayekpam, Manoharmayum Birkumar Sharma

**Affiliations:** 1 Department of Radiodiagnosis, Regional Institute of Medical Sciences (RIMS), Imphal, IND; 2 Department of General Surgery, Regional Institute of Medical Sciences (RIMS), Imphal, IND

**Keywords:** 3 tesla mri, bi-rads, breast cancer, dce-mri, diffusion-weighted imaging, multiparametric mri

## Abstract

Background

Breast cancer is the most common malignancy affecting women globally. Early and accurate characterization of breast lesions is essential for the accurate diagnosis of breast carcinoma, reducing unnecessary biopsies and improving overall survival. Multiparametric magnetic resonance imaging (MRI) at 3 Tesla (3T), incorporating dynamic contrast-enhanced MRI (DCE-MRI), diffusion-weighted imaging (DWI), and proton MR spectroscopy (MRS), has emerged as a promising diagnostic modality for breast lesion characterization.

Objective

This study aimed to evaluate the diagnostic accuracy of multiparametric 3T MRI in differentiating benign and malignant breast lesions using the Breast Imaging Reporting and Data System (BI-RADS) classification.

Methods

This hospital-based cross-sectional study was conducted in the Department of Radiodiagnosis in collaboration with the Department of General Surgery at the Regional Institute of Medical Sciences, Imphal, India, from January 2021 to October 2022. A total of 62 female patients aged above 18 years presenting with breast lesions were included. All patients had previously undergone ultrasonography examination for palpable breast lesions, either within the Department of Radiodiagnosis or at outside centers.

Patients demonstrating positive or suspicious findings on ultrasonography were subsequently referred for multiparametric MRI of the breast. Ultrasonography findings were obtained from available reports; however, no statistical comparison between ultrasound and MRI findings was performed, as this was beyond the scope of the study.

MRI findings were categorized according to BI-RADS criteria as per the American College of Radiology guidelines. Histopathological evaluation served as the reference standard. Diagnostic performance parameters were calculated. Statistical analysis was performed using IBM SPSS Statistics for Windows, Version 21.0 (IBM Corp., Armonk, New York, United States).

Results

Among 62 participants, the mean age of the participants was 51.1±10.2 years (range: 30-80 years). The most common presenting complaint was a breast lump, 62 (100%), followed by axillary swelling, 56 (90%). On MRI, most lesions demonstrated heterogeneous enhancement, 39 (62.7%), and irregular margins, 34 (55.2%). Type III kinetic curve (washout pattern) was the most frequent, 56 (89.5%). Diffusion restriction was consistently observed in malignant lesions. Increased choline peaks were observed in lesions where MR spectroscopy was performed. Histopathological evaluation revealed 57 malignant and five benign lesions. The diagnostic performance of multiparametric MRI using the BI-RADS scoring system demonstrated a sensitivity of 93%, a specificity of 60%, a positive predictive value (PPV) of 96.4%, and a negative predictive value (NPV) of 42.9%. A statistically significant association was observed between MRI findings and histopathological diagnosis (Fisher's exact test; p=0.008). The area under the receiver operating characteristic (ROC) curve was 0.968.

Conclusion

Multiparametric 3T MRI is a highly sensitive imaging modality for the characterization of breast lesions, with moderate specificity. Its use improves diagnostic accuracy and demonstrates a strong correlation with histopathological findings in patients with suspicious breast lesions.

## Introduction

Breast cancer is the most commonly diagnosed malignancy among women worldwide. According to GLOBOCAN 2018, the global incidence rate was 24.7 per 100,000 women, making it the leading cancer in females, with a mortality rate of 13.4 per 100,000. In India, GLOBOCAN 2018 reported 162,468 new breast cancer cases out of a total of 1,157,294 newly diagnosed cancers. Breast cancer also accounted for the highest number of cancer-related deaths (87,090) among both genders.

Epidemiological trends indicate a rising incidence of breast cancer in regions such as Manipur, with an annual percent change (APC) of 3.78% between 2005 and 2014. The lifetime risk of developing breast cancer is higher in urban populations (one in 22 women) compared to rural populations (one in 60 women) [1]. While the incidence of breast cancer is greater in developed countries, mortality rates are relatively higher in less developed regions. Furthermore, improved survival and increasing incidence have contributed to a growing global prevalence of breast cancer [2].

Notably, more than 50% of patients present at advanced stages (stages III and IV), where treatment outcomes are less favorable despite aggressive management [3]. Therefore, early detection remains a critical factor in improving patient outcomes and reducing mortality associated with breast cancer.

B-mode ultrasonography (USG) is a highly sensitive modality for the detection of breast lesions; however, its specificity for lesion characterization remains limited, as reported in several studies. This limitation is largely due to the subjective and operator-dependent nature of image acquisition and interpretation, which can lead to variability in assessing lesion features.

The introduction of the Breast Imaging Reporting and Data System (BI-RADS) lexicon has reduced such variability by standardizing reporting and categorizing lesions based on the probability of malignancy. Lesions classified as BI-RADS categories 4 and 5 are considered suspicious and are more likely to represent malignancy. Nevertheless, the use of BI-RADS criteria alone still results in a considerable number of false-positive findings, leading to increased biopsy rates with relatively low positive cancer detection rates (approximately 10-30%).

False-positive biopsy rates based on USG BI-RADS assessment have been reported to be as high as 66%, with many lesions ultimately proven benign on histopathology. This contributes to unnecessary healthcare costs and increased patient anxiety in routine breast cancer screening programs [4].

Therefore, there is a need for an additional diagnostic modality to improve the differentiation between benign and malignant breast masses. Such an approach would help reduce unnecessary biopsies, lower costs, shorten waiting times, and alleviate patient anxiety. In clinical practice, magnetic resonance imaging (MRI) is widely used for characterizing tumor phenotypes and for monitoring treatment response, providing valuable complementary information in breast lesion evaluation [5].

Conventional imaging modalities for breast evaluation, such as mammography and USG, are associated with a relatively high rate of false-positive findings after biopsies [6]. Mammography is an important screening modality due to its ability to detect microcalcifications, particularly in early ductal carcinoma in situ (DCIS), and is especially valuable in non-palpable lesions. Also, ultrasound and mammography have their limitations for lesion detection in dense breast. To address these limitations, additional imaging modalities are required to improve diagnostic specificity and reduce unnecessary interventions. MRI, particularly multiparametric MRI, has emerged as a valuable problem-solving tool in breast imaging by providing both morphological and functional information. MRI demonstrates superior sensitivity compared to conventional imaging modalities, particularly in dense breast tissue.

With advancements in imaging technology, high-field strength MRI, particularly at 3 Tesla (3T), has become increasingly available in clinical practice. Its use in breast cancer evaluation is expanding due to advantages such as improved spatial resolution, higher signal-to-noise ratio, and reduced imaging time, thereby enhancing diagnostic accuracy [7].

Emerging evidence suggests that multiparametric imaging, which integrates multiple functional MRI parameters, provides comprehensive insights into the key biological characteristics of breast cancer [8]. These include neoangiogenesis, cellularity, tumor microenvironment, metabolic activity, receptor status, tissue pH, and oxygenation [9].

Such detailed information cannot be fully captured by a single technique, such as contrast-enhanced MRI (CE-MRI) alone. Therefore, the use of multiparametric MRI enhances diagnostic accuracy and improves the evaluation of treatment response, particularly in patients undergoing neoadjuvant therapy [10].

Dynamic contrast-enhanced (DCE) MRI is widely utilized to improve the specificity of MRI in the characterization of breast lesions. It enables the analysis of time-signal intensity curves, which provide valuable information for predicting the likelihood of malignancy [11].

Diffusion-weighted imaging (DWI) has recently been incorporated into breast MRI protocols, offering the potential to further enhance diagnostic specificity. DWI is a functional MRI technique that evaluates the microdiffusion of water molecules within intracellular and extracellular spaces [12].

Recent studies have demonstrated its utility in differentiating benign from malignant tumors through the measurement of apparent diffusion coefficient (ADC) values [13]. Quantitative assessment of ADC has shown promising results in tumor characterization and in estimating tumor aggressiveness [14]. However, despite its value as a biomarker for detecting malignancy, ADC has not proven to be a reliable prognostic indicator in breast cancer [15]. Studies have not demonstrated a significant correlation between ADC values and established prognostic factors such as tumor size, lymph node involvement, and histological grade [16].

In addition, proton MR spectroscopic imaging provides insight into tumor metabolism by detecting choline levels, a marker of increased cellular turnover that is typically elevated in breast cancer.

Fine needle aspiration cytology (FNAC) is a well-established and reliable method for diagnosing malignancy in suspicious breast lesions, demonstrating good sensitivity and positive predictive value (PPV) when performed by an experienced cytopathologist [17]. In selected cases, particularly early operable breast cancers suitable for breast-conserving surgery, treatment decisions can be made based solely on FNAC findings. Owing to its cost-effectiveness and minimally invasive nature, FNAC is widely used as a primary preoperative diagnostic tool for evaluating breast lumps, and it can help avoid unnecessary surgical procedures [18].

With improvements in its diagnostic accuracy, the use of intraoperative frozen-section histology has declined significantly. However, FNAC is still associated with a higher rate of diagnostic errors compared to histopathology [19]. In recent years, core needle biopsy has increasingly replaced FNAC as the preferred modality for breast cancer diagnosis. Core biopsy provides superior histopathological assessment, including accurate lesion characterization and tumor grading, and has demonstrated higher sensitivity and specificity in multiple studies, including those by Baltzer and Dietzel [20].

This cross-sectional hospital-based study was conducted in the Department of Radiodiagnosis at the Regional Institute of Medical Sciences, Imphal, India, to evaluate the multiparametric 3T MRI findings of various breast lesions. The objective of this study was to evaluate the multiparametric MRI findings in various breast lesions and to assess the diagnostic accuracy of multiparametric 3T MRI using the BI-RADS classification system.

## Materials and methods

Study design and setting

This evidence-based cross-sectional study was conducted in the Department of Radiodiagnosis in collaboration with the Department of General Surgery at the Regional Institute of Medical Sciences, Imphal, India. Data is collected at a single point of time.

Study duration

The study was carried out over a period of two years, from January 2021 to October 2022.

Study population

The study included female patients presenting with breast lesions who attended the Department of General Surgery and were subsequently referred to the Department of Radiodiagnosis for breast MRI during the study period. All patients meeting the inclusion criteria were enrolled.

Inclusion criteria

Female patients aged above 18 years presenting with breast lesion(s), irrespective of the number, duration, location, or laterality, and who provided informed consent were included in the study.

Exclusion criteria

Patients who were post-operative, post-radiation, or post-FNAC/biopsy were excluded. Additionally, patients with isolated ductal ectasia without a focal lesion were not included. Cases categorized as BI-RADS 0 (incomplete evaluation requiring additional imaging), BI-RADS 1 (negative study), and BI-RADS 6 (biopsy-proven malignancy) were also excluded.

Sample size and sampling

The sample size was determined based on previously reported sensitivity (~90%) of breast MRI. Considering a 95% confidence interval and allowable error, the minimum sample size was calculated as 57. After accounting for a 10% non-response rate, 62 patients were included. 

The sensitivity of multiparametric MRI for accurately diagnosing breast lesions has been reported to be 90% in a study by Radhakrishna et al. [21]. Additionally, the prevalence of breast lumps in the female population is approximately 15.3%, as reported by Das et al. [22]. With a precision of 95% and a margin of error (L) of 2 (L=2/1.96=1.020), the calculated sample size was 57. After accounting for a 10% non-response rate, the final sample size was increased to 62.

Study variables

The independent variables included age, parity status, menstrual status, presenting complaints, and duration of symptoms, while the dependent variables comprised the radiological profile of breast lesions as evaluated by B-mode USG and multiparametric 3T MRI using the BI-RADS classification system.

MRI findings

MRI findings were evaluated based on DCE-MRI, DWI, and proton MR spectroscopy.

Pathological findings

Histopathological examination using core needle biopsy served as the reference standard for diagnosis.

This methodology was designed to comprehensively evaluate the diagnostic performance of multiparametric 3T MRI in the characterization of breast lesions and its correlation with conventional imaging and pathological findings.

Study tools

MRI examinations were conducted using a Siemens 3T MRI system with a phased-array breast coil. The multiparametric MRI protocol included 2D T2-weighted imaging, DWI, DCE-MRI, and MR spectroscopic imaging.

Outcome variables

The outcome measures included sensitivity, specificity, PPV, and negative predictive value (NPV), which were used to assess the diagnostic accuracy of multiparametric MRI in the detection and evaluation of breast lesions.

Data collection and imaging protocol

Informed consent was obtained from all patients who fulfilled the inclusion criteria prior to participation. Detailed clinical information, including age, sex, marital status, occupation, religion, address, presenting complaints with duration, parity, and findings from general and systemic examinations, along with relevant investigations, were collected from the Department of General Surgery. Patients were adequately informed about the procedures, and confidentiality was strictly maintained throughout the study.

All patients with suspected or detected breast lesions referred to the Department of Radiodiagnosis of Regional Institute of Medical Sciences, Imphal, India, who already underwent ultrasonographic evaluation inside the hospital or outside centers were enrolled in the study.

All lesions identified or suspected based on previous ultrasonography findings were further evaluated using multiparametric 3T MRI. Lesions were categorized according to the BI-RADS classification system as recommended by the American College of Radiology (ACR), ranging from category 0 (incomplete) to category 6 (biopsy-proven malignancy). For the purpose of this study, BI-RADS categories 0, 1, and 6 were excluded from the analysis.

MRI protocol

All MR examinations were performed with the patient in the prone position using a 3T MRI system (Skyra, Erlangen, Germany) equipped with a dedicated four-channel breast coil (In Vivo, Orlando, Florida, United States). In premenopausal women, imaging was scheduled during the second week of the menstrual cycle to minimize hormonal influences on breast parenchyma.

The MRI protocol was acquired in the following sequence: (a) For T2-weighted imaging, a turbo spin-echo sequence with fat suppression was obtained (TR/TE/TI: 4800/61/230 ms; field of view: 340 mm; slice thickness: 4 mm with 34 slices; matrix: 314×320; acquisition time: 2 minutes 26 seconds). (b) For DWI, double-refocused, single-shot echo-planar imaging with inversion recovery fat suppression was performed (TR/TE/TI: 13700/83/220 ms; field of view: 340×117 mm; slice thickness: 3.5 mm with 40 slices; matrix: 192×64 with 50% oversampling; b-values: 50 and 850 s/mm²; acquisition time: 3 minutes 19 seconds). (c) For DCE-MRI, a split dynamics protocol combining high spatial and temporal resolution was used. This included T1-weighted volume interpolated breath-hold examination (VIBE) sequences (TR/TE: 3.61/1.4 ms; field of view: 320 mm; 72 slices; 1.7-mm isotropic resolution; matrix: 192×192; acquisition time: 13.2 seconds per volume; total acquisition time: 15 minutes 20 seconds) and T1-weighted turbo fast low-angle shot (FLASH) 3D sequences with selective water excitation (TR/TE: 877/3.82 ms; field of view: 320 mm; 96 slices; 1-mm isotropic resolution; matrix: 320×134; acquisition time: 2 minutes).

All patients received a single intravenous dose of contrast agent, gadoterate meglumine (Dotarem; Guerbet, Roissy, France), administered as a bolus at 0.1 mmol/kg body weight at a rate of 4 mL/s, followed by a 20 mL saline flush using a power injector (Spectris Solaris EP; Medrad, Pittsburgh, Pennsylvania, United States). Contrast administration was initiated 75 seconds after the start of the first coronal T1-weighted VIBE sequence.

The total examination time for the multiparametric MRI protocol was approximately 34 minutes.

DCE-MRI of the breast

DCE-MRI findings were interpreted using descriptors from the ACR MRI BI-RADS lexicon to differentiate benign from malignant contrast-enhancing lesions. For mass lesions, the following features were evaluated: shape (round, oval, lobulated, or irregular), margins (smooth, irregular, or spiculated), internal enhancement pattern (homogeneous or heterogeneous), and enhancement kinetics. Kinetic curves were categorized as type I (persistent enhancement), type II (initial rapid enhancement with plateau), and type III (initial rapid enhancement with washout).

For non-mass-like enhancement (NMLE), assessment included distribution patterns (focal, regional, multiple regions, segmental, ductal, linear, or diffuse), internal enhancement characteristics (homogeneous, heterogeneous, clumped, or stippled), and symmetry. Based on these parameters, a final BI-RADS category was assigned to estimate the probability of malignancy.

DWI

DWI analysis was performed using ADC values. Based on the receiver operating characteristic (ROC) curve analysis, an optimal ADC threshold of 1.25×10⁻³ mm²/s was used to differentiate benign from malignant lesions. Lesions with ADC values ≥1.25×10⁻³ mm²/s were classified as benign, while those with values <1.25×10⁻³ mm²/s were considered malignant.

Proton MR spectroscopy

Proton MR spectroscopy was used to evaluate tumor metabolism by measuring the choline signal-to-noise ratio (Cho-SNR). A threshold value of 2.6 was applied, with lesions classified as malignant if the SNR was ≥2.6 and benign if <2.6. However, MR spectroscopy was not routinely performed as part of the standard DCE-MRI breast protocol.

Data collection

Informed written consent was obtained from all participants prior to inclusion in the study. Demographic and clinical data, including age, sex, educational status, occupation, history of present and past illness, personal and family history, comorbidities, symptomatology, and clinical examination findings, were recorded.

Statistical analysis

Data were entered and analyzed using IBM SPSS Statistics for Windows, Version 21.0 (IBM Corp., Armonk, New York, United States). Microsoft Excel (Microsoft Corporation, Redmond, Washington, United States) was used for generating graphs and tables. Descriptive statistics were presented as frequencies, proportions, percentages, means, and standard deviations. Inferential analysis was performed using the chi-squared test to assess associations between categorical variables.

Ethical considerations

Informed written consent was obtained from all participants. Patient confidentiality and privacy were strictly maintained throughout the study. Ethical approval was obtained from the Research Ethics Board of the Regional Institute of Medical Sciences (RIMS) (approval number: A/206/REB-Comm(SP)/RIMS/2015/794/136/2020).

## Results

Age class distribution

A total of 62 women participated in the study. The majority, 23 (37.3%), belonged to the age group of 50-60 years, followed by approximately 20 (31.3%) having 40-50 years of age, 10 (16.4%) above 60 years old, and nine (15%) 30-40 years of age. The mean age of the participants was 51.1±10.2 years (range: 30-80 years).

Sociodemographic profile

The majority of female patients were homemakers, approximately 45 (71.8%), followed by patients who were employed, 17 (28.2%).

Parity distribution

Table 1 shows the distribution of parity status among the study participants. The majority of patients were multiparous, 51 (82.3%), while a smaller proportion were nulliparous, 11 (17.7%).

**Table 1:** Parity distribution (N=62) n: actual number of patients; N: total number of participants

Parity status	Benign (n=5)	Malignant (n=57)	Total (N=62)
Nulliparous	1	10	11
Multiparous	4	47	51

A higher proportion of malignant lesions was observed among multiparous women (47/51) compared to nulliparous women (10/11). Fisher's exact test did not demonstrate a statistically significant association between parity status and malignancy (p>0.05), possibly due to the small sample size and predominance of malignant cases in the study population.

Distribution of lesions according to menopausal status

The majority of patients fall under the menopause group, constituting 50 (80.5%) of the study population, followed by premenopausal women, constituting 12 (19.5%).

Distribution of duration of illness

Duration of illness varies from a minimum of six days to a maximum of seven months. Out of all participants, 22 (35.8%) were ill for a duration of 3-4 months, followed by 19 (31.4%) who were ill for >4 months, 17 (26.8%) up to 1-2 months, and four (6%) <1 month, as illustrated in Table 2.

**Table 2:** Distribution of duration of illness n: number of patients; %: percentage of participants

Duration of illness	n (%)
<1 month	4 (6%)
1-2 months	17 (26.8%)
3-4 months	22 (35.8%)
>4 months	19 (31.4%)

Distribution of chief complaints

The most common presenting complaint was breast lump, 62 (100%), followed by axillary swelling, 56 (90%), nipple discharge, 18 (30%), pain, 12 (19%), nipple retraction, nine (15%), skin change, six (10%), and fever, one (2%). Several patients had overlapping presenting symptoms as illustrated in Table 3.

**Table 3:** Distribution of chief complaints n: number of patients; %: percentage of participants

Chief complaints	n (%)
Lump	62 (100%)
Axillary swelling	56 (90%)
Pain	12 (19%)
Nipple discharge	18 (30%)
Nipple retraction	9 (15%)
Fever	1 (2%)
Skin change	6 (10%)

Distribution of the shape of breast lesions

The majority, 38 (61%), of breast lesions included in our study were oval in shape, followed by irregular, 17 (28%), and round, seven (12%), as illustrated in Table 4.

**Table 4:** Distribution of the shape of breast lesions N: number of patients; %: percentage of participants

Shape of breast lesions	n (%)
Oval	38 (61%)
Irregular	17 (28%)
Round	7 (12%)

Distribution of the size of breast lesions in MRI

The majority of cases had lesions of size ranging from 2×2 cm to <3×3 cm, 24 (37.3%), followed by <2×2 cm, 15 (23.8%), >3×3 cm to 4×4 cm, 12 (20.8%), and >4×4 cm, 11 (17.9%), as illustrated in Table 5.

**Table 5:** Distribution of the size of breast lesions in MRI MRI: magnetic resonance imaging; n: number of patients; %: percentage of participants

MRI findings of breast lesions in size	n (%)
<2×2 cm	15 (23.8%)
2×2 cm to <3×3 cm	24 (37.3%)
>3×3 cm to 4×4 cm	12 (20.8%)
>4×4 cm	11 (17.9%)

Distribution of enhancement characteristics

The majority, 39 (62.7%), of breast lesions showed heterogeneous enhancement, and 23 (37.3%) of cases showed homogenous enhancement. Analysis of enhancement characteristics showed a significant association with malignancy, particularly in lesions exhibiting heterogeneous enhancement.

Distribution of the margins of breast lesions in MRI

The majority of breast lesions showed irregular margins, 34 (55.2%), followed by speculated, 19 (29.8%), and lobulated, nine (15%). Lesions with irregular and spiculated margins were more suspicious for malignancy.

Distribution of the consistency of breast lesions

The solid lesions constituted 57 (92.5%), followed by mixed solid-cystic lesions, three (4.5%), and cystic lesions, two (3%). There was a significant correlation between consistency pattern and malignancy of breast lesions. Analysis showed solid-appearing lesions in MRI to have a higher degree of correlation with malignancy.

Internal enhancement pattern of non-mass enhancement (NME) of breast lesions

The commonly encountered enhancement pattern for the NME type of breast lesions was the heterogeneous type, 39 (62.7%), followed by homogeneous enhancement, 23 (37.3%). None of the cases showed clumped or cluster pattern enhancement, 0 (0%) each, in our study, as illustrated in Table 6.

**Table 6:** Internal enhancement pattern of NME of breast lesion NME: non-mass enhancement; n: number of participants; %: percentage of participants

NME	n (%)
Heterogeneous	39 (62.7%)
Homogeneous	23 (37.3%)
Clumped	0 (0%)
Cluster	0 (0%)

Kinetic curve (DCE-MRI) of breast lesions

Kinetic curves in DCE-MRI were mostly type III, i.e., 55 (89.5%), followed by type II, which was in the plateau phase of five (7.5%), and type I, two (3%). A statistically significant association was observed between type III kinetic curves and malignancy. 

BI-RADS score

In the present study comprising 62 participants, BI-RADS category 4 lesions constituted the majority, accounting for 54 cases (87.1%). This was followed by BI-RADS category 5 lesions in five cases (8.06%), BI-RADS category 3 lesions in two cases (3.2%), and BI-RADS category 2 lesions in one case (1.61%).

Correlation of BI-RADS categories with histopathological diagnosis

A strong correlation was observed between BI-RADS categories and histopathological diagnosis, with BI-RADS 4 and 5 lesions showing a high likelihood of malignancy (Table 7), confirming the diagnostic utility of the BI-RADS classification system in our study.

**Table 7:** Correlation of BI-RADS categories with histopathological diagnosis (N=62) BI-RADS: Breast Imaging Reporting and Data System; N: total number of participants

BI-RADS category	Benign	Malignant	Total
2	1	0	1
3	2	0	2
4	2	52	54
5	0	5	5

Representative imaging findings

Representative multiparametric MRI findings of BI-RADS 2, 4, and 5 lesions, along with representative histopathological correlation, are illustrated (Figures 1-13).

**Figure 1:**
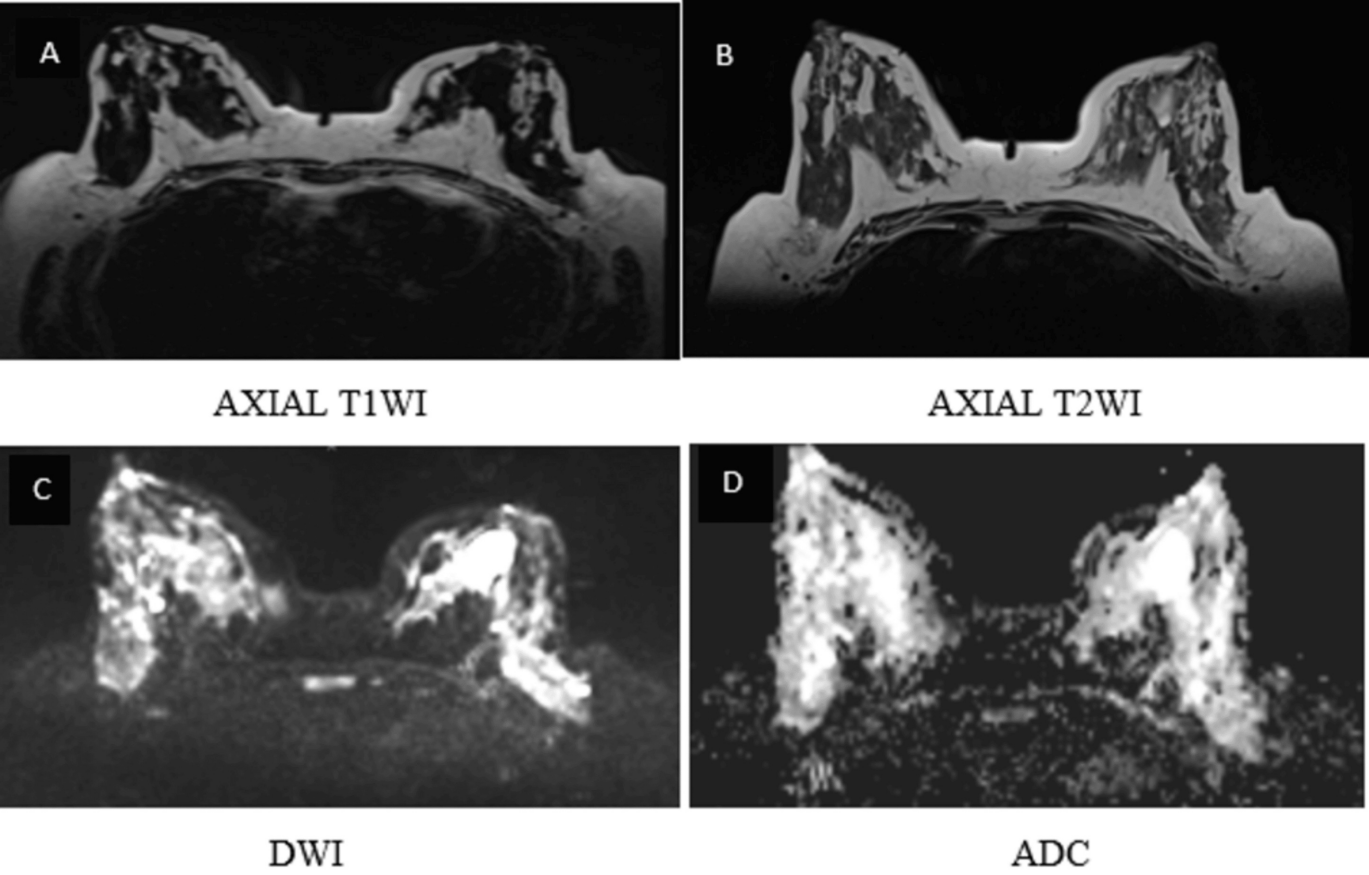
BI-RADS 2 lesion shows a cyst in the left breast, hypointense on T1WI and hyperintense on T2WI (A, B). DWI demonstrates no diffusion restriction (C, D) BI-RADS: Breast Imaging Reporting and Data System; T1WI: T1-weighted image; T2WI: T2-weighted image; DWI: diffusion-weighted imaging; ADC: apparent diffusion coefficient

**Figure 2:**
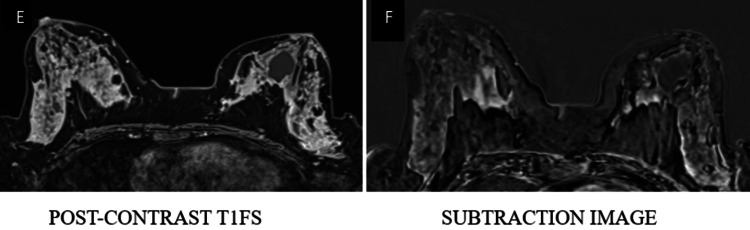
Post-contrast T1FS and subtraction image (E, F) demonstrate no post-contrast enhancement T1FS: T1-weighted with fat saturation

**Figure 3:**
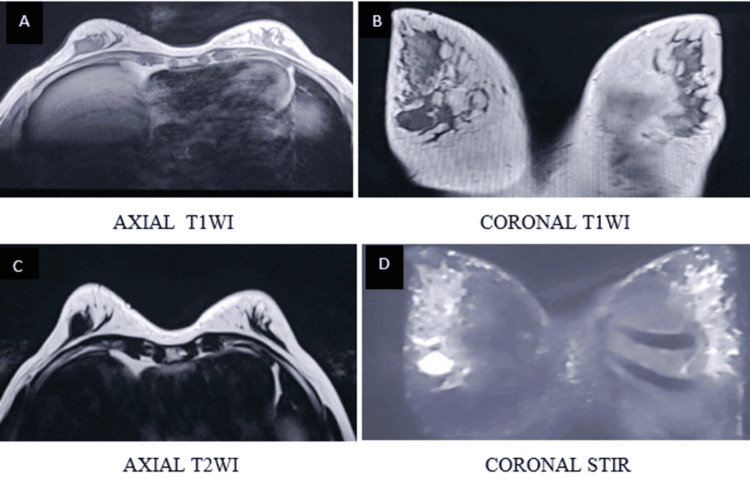
A well-defined oval-shaped mass lesion in the lower outer quadrant of the right breast appears isointense on axial T1WI (A, B), isointense on axial T2WI (C), and hyperintense on coronal STIR images (D) T1WI: T1-weighted image; T2WI: T2-weighted image; STIR: short TI inversion recovery

**Figure 4:**
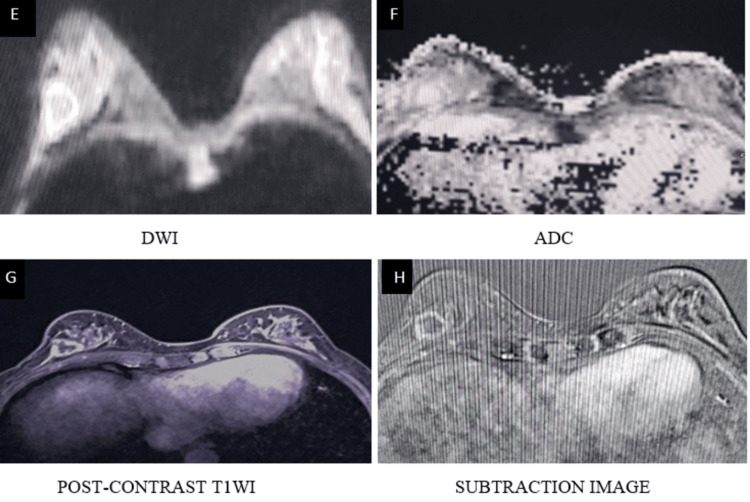
The lesion shows peripheral rim diffusion restriction (E, F) and rim enhancement (G, H) on post-contrast imaging DWI: diffusion-weighted imaging; ADC: apparent diffusion; T1WI: T1-weighted image

**Figure 5:**
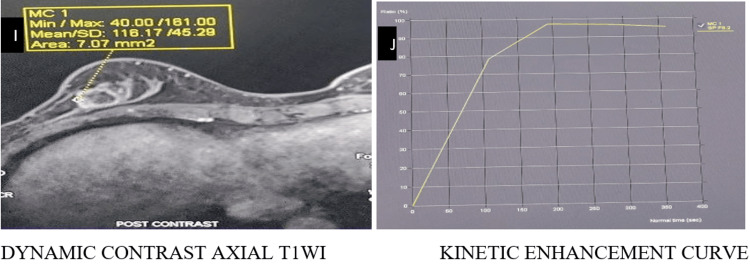
Type II intensity curve shows rapid enhancement and plateau (I, J) categorized as BI-RADS 4, and histopathological examination confirmed infiltrating ductal carcinoma BI-RADS: Breast Imaging Reporting and Data System; T1WI: T1-weighted image

**Figure 6:**
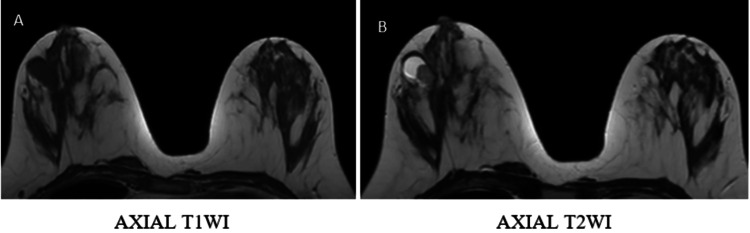
BI-RADS 4 lesion shows a solid-cystic mass in the outer quadrant of the right breast. The lesion appears isointense on T1WI and demonstrates a hyperintense cystic component with intermediate to mildly hyperintense signal in the solid component on T2WI (A, B) BI-RADS: Breast Imaging Reporting and Data System; T1WI: T1-weighted image; T2WI: T2-weighted image

**Figure 7:**
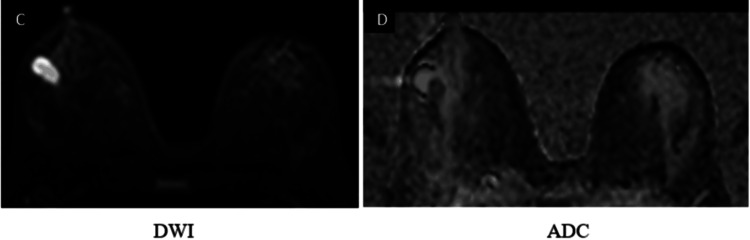
DWI and ADC maps (C, D) demonstrate marked diffusion restriction in the solid component of the lesion DWI: diffusion-weighted imaging; ADC: apparent diffusion

**Figure 8:**
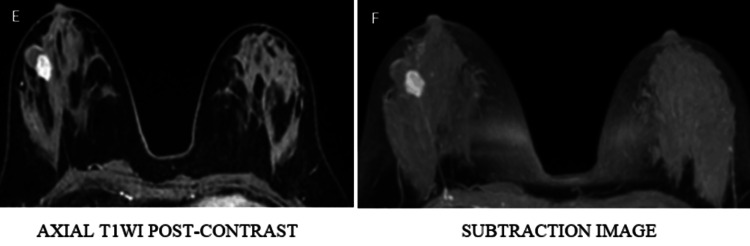
Dynamic post-contrast imaging (E, F) demonstrates intense enhancement of the solid component T1WI: T1-weighted image

**Figure 9:**
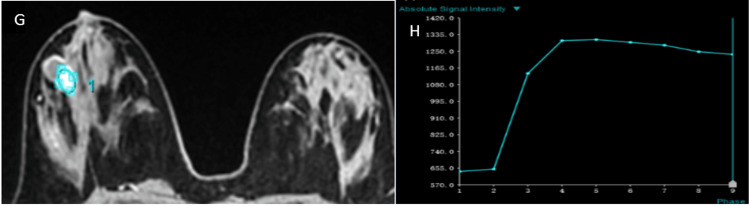
Type II (indeterminate) kinetic curve (G, H)

**Figure 10:**
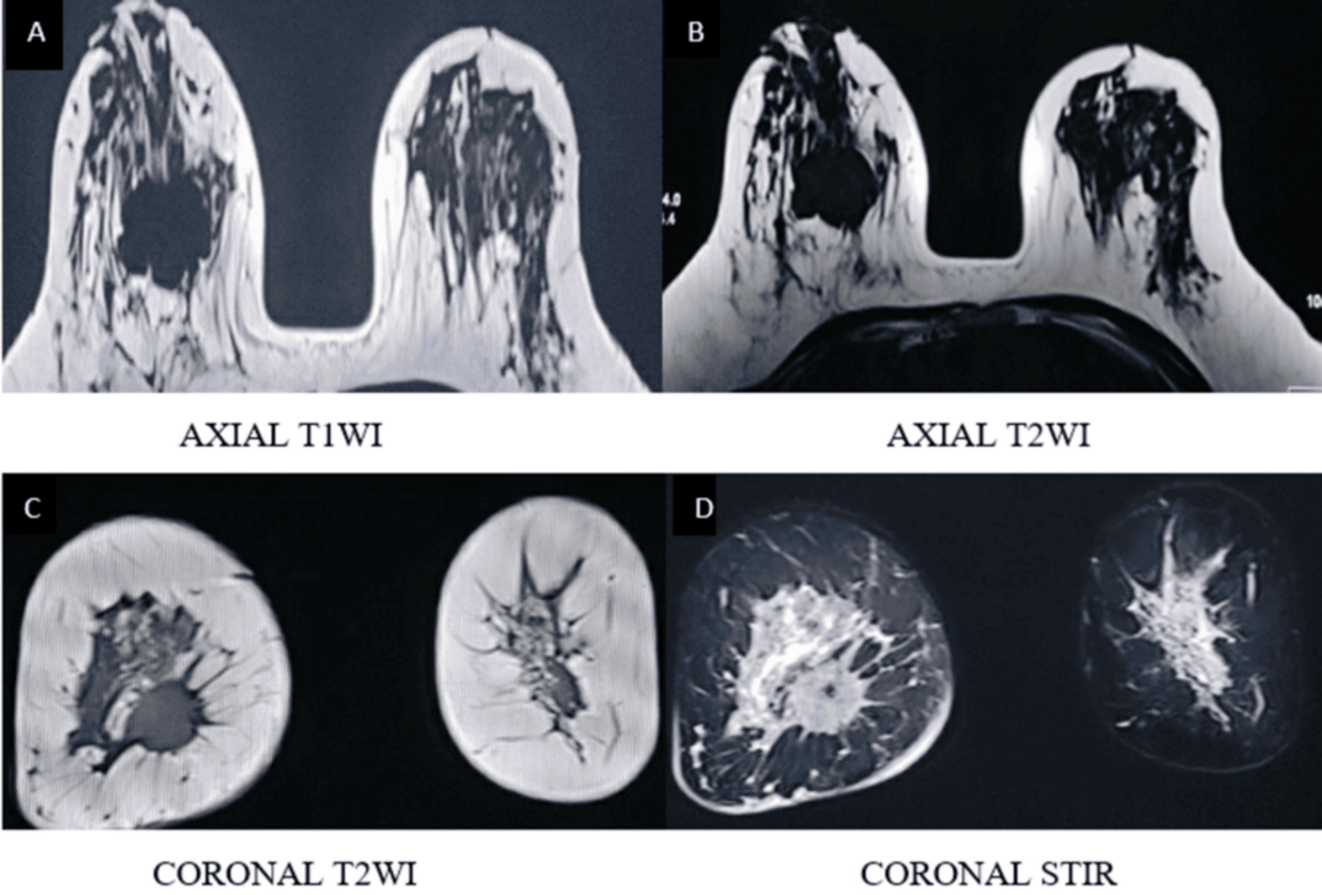
A spiculated mass lesion in the lower inner quadrant of the right breast appears isointense on T1WI and T2WI (A, B, C) and hyperintense on STIR images (D) T1WI: T1-weighted image; T2WI: T2-weighted image; STIR: short TI inversion recovery

**Figure 11:**
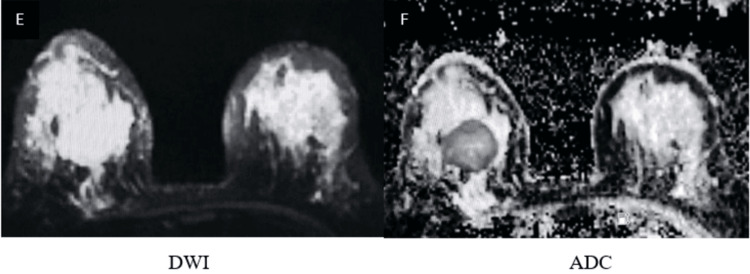
Round to oval shape lesion in the right breast demonstrates marked heterogenous peripheral diffusion restriction on DWI (E, F) DWI: diffusion-weighted imaging; ADC: apparent diffusion

**Figure 12:**
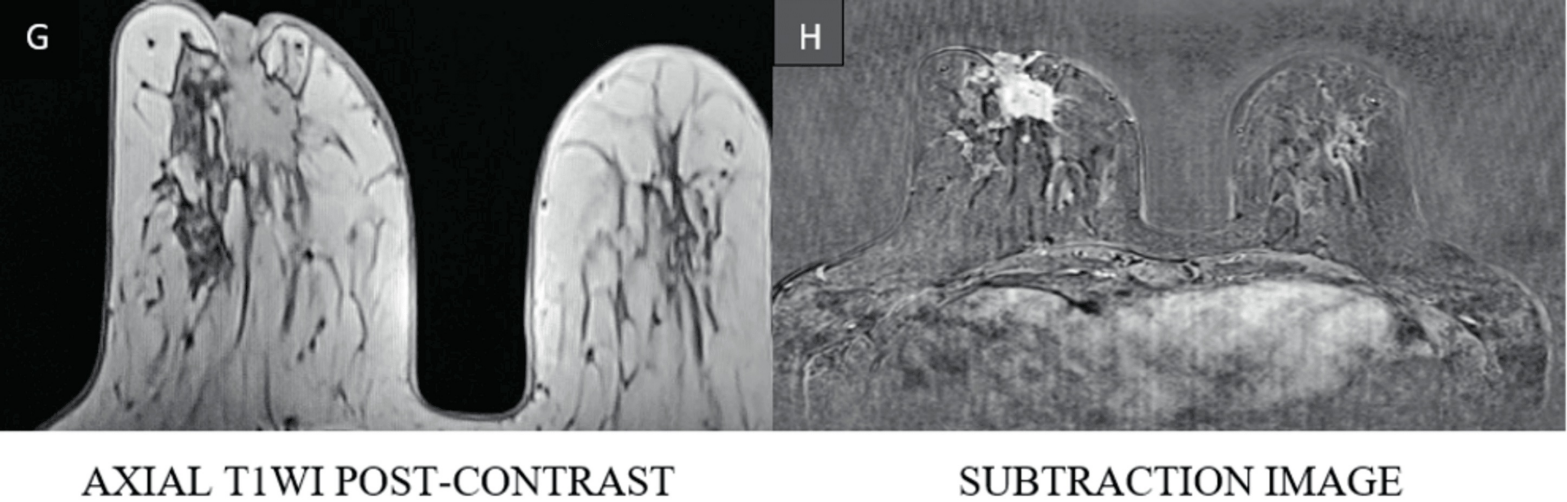
Right breast lesion demonstrates avid heterogeneous enhancement on post-contrast imaging (G, H) T1WI: T1-weighted image

**Figure 13:**
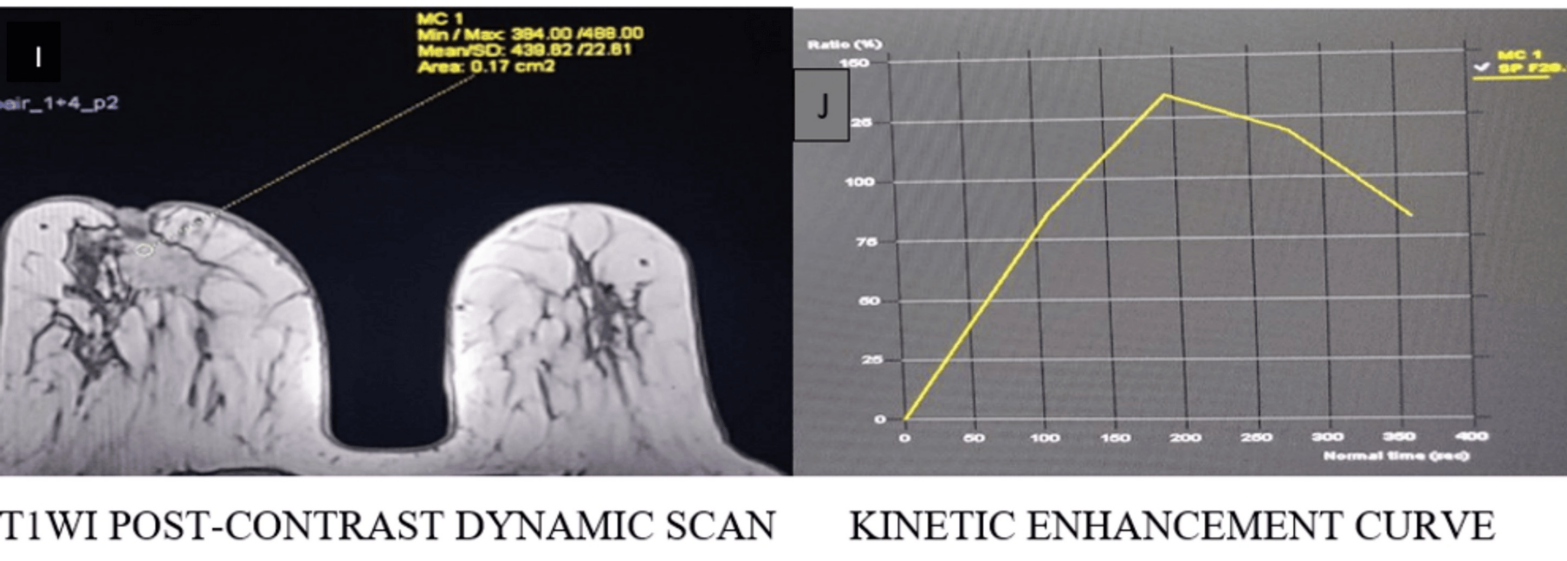
Type III kinetic curve shows rapid initial enhancement with washout (I, J) categorized as BI-RADS 5, and histopathological examination confirmed infiltrating ductal carcinoma BI-RADS: Breast Imaging Reporting and Data System; T1WI: T1-weighted image

**Figure 14:**
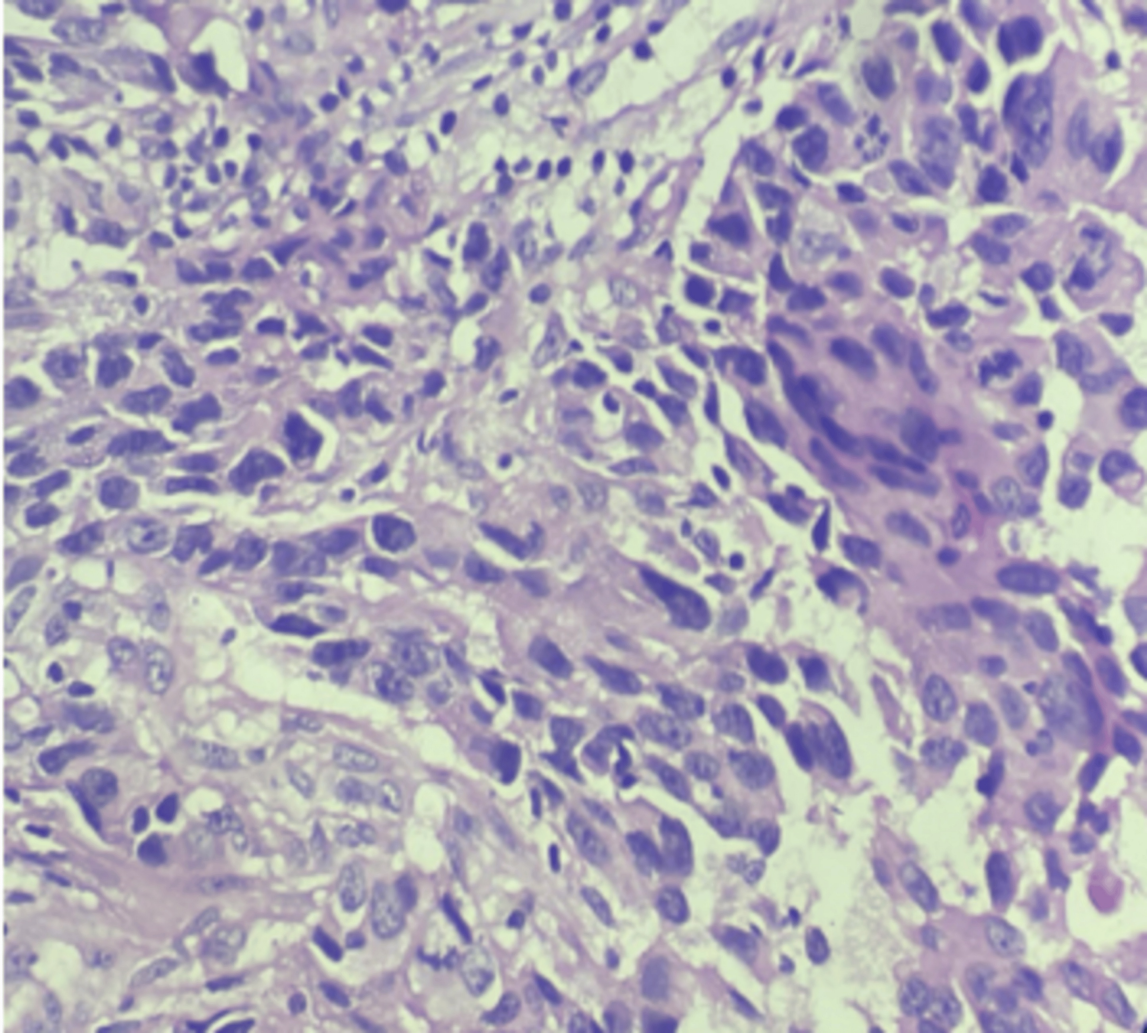
Representative histopathological examination of one of the breast lesions

The photomicrograph of the section studied shows moderately pleomorphic malignant cells arranged in sheets and nests with an increased nuclear-to-cytoplasmic ratio. The nuclei are oval to elongated and vesicular to hyperchromatic, with many cells exhibiting prominent nucleoli and a moderate amount of eosinophilic cytoplasm. Tumor-infiltrating lymphocytes (TILs) are also noted, as shown in Figure 14. These features are consistent with infiltrating ductal carcinoma, no special type (IDC-NST).

MRI diagnosis demonstrated 53 true positives, 2 false positives, 4 false negatives, and 3 true negatives (Table 8). These values were used to calculate sensitivity, specificity, PPV, and NPV.

**Table 8:** Correlation of MRI findings with histopathology (N=62) MRI: magnetic resonance imaging; N: total number of participants; TP: true positive; TN: true negative; FP: false positive; FN: false negative

MRI diagnosis	Malignant	Benign	Total
Malignant (positive)	53 (TP)	2 (FP)	55
Benign (negative)	4 (FN)	3 (TN)	7
Total	57	5	62

Diagnostic accuracy of MRI in breast lesions

The diagnostic performance of multiparametric MRI using the BI-RADS scoring system demonstrated a sensitivity of 93%, a specificity of 60%, a PPV of 96.4%, and an NPV of 42.9%. A statistically significant association was observed between MRI findings and histopathological diagnosis (Fisher's exact test; p=0.008). The relatively lower specificity observed in this study may be attributed to the high prevalence of malignant lesions and the limited number of benign cases, which can influence specificity estimates. Additionally, MRI is inherently a highly sensitive modality, often at the expense of specificity, leading to increased false-positive findings as illustrated in Table 9.

**Table 9:** Diagnostic accuracy of MRI in breast lesions MRI: magnetic resonance imaging; n: number of patients; %: percentage of participants

Sensitivity	Specificity	Positive predictive value	Negative predictive value	P-value
93%	60%	96.4%	42.9%	0.008

Distribution of pathological diagnoses of breast lesions

In our study, malignant breast lesions constituted the majority of the study population when compared to benign breast lesions. The most commonly encountered malignant lesion was infiltrating ductal carcinoma, followed by invasive carcinoma, not otherwise classified, inflammatory carcinoma, and invasive lobular carcinoma. Of all the benign lesions, the most common pathology is fibroadenoma, followed by fibrocystic breast disease, as illustrated in Table 10.

**Table 10:** Distribution of pathological diagnosis of breast lesions n: number of patients; %: percentage of participants

Benign lesions (n=5)	Frequency
Granulomatous mastitis	1
Breast abscess	1
Fibroadenoma	2
Fibrocystic breast disease	1
Malignant lesions (n=57)	Frequency
Infiltrating ductal carcinoma	30
Invasive lobular carcinoma	2
Invasive carcinoma, not otherwise classified	18
Inflammatory carcinoma	7

ROC curve

**Figure 15:**
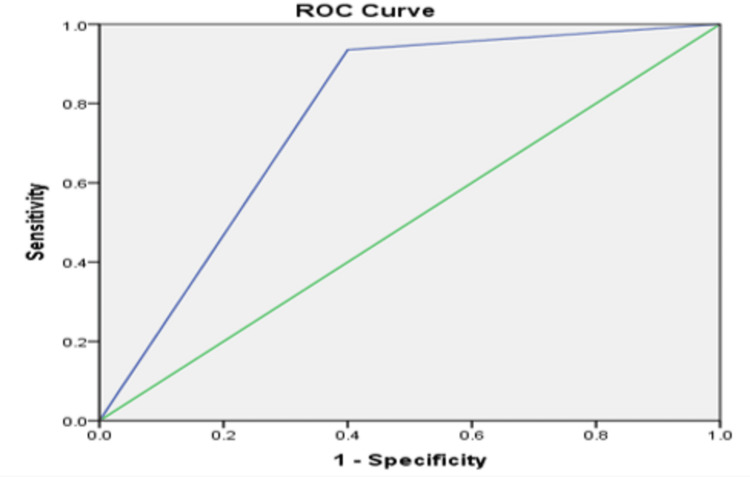
ROC curve shows the sensitivity and specificity of MRI in breast lesions ROC: receiving operating characteristics; MRI: magnetic resonance imaging

Figure 15 shows an area under the ROC curve (AUC) of 0.968, indicating excellent diagnostic performance.

## Discussion

In the present study of 62 patients, the mean age was 51.1±10.2 years, with an age range of 30-80 years. This finding is comparable to previous studies, including those by Pinker et al. (mean age: 52 years), Bitencourt et al. (mean age: 47.8 years), and Tsukada et al. (mean age: 56 years) [23-26]. Most patients in the current study were in the fourth and fifth decades of life. Similar observations were reported by Sathwara et al. [27], with a mean age of 49 years, and by Tawfeeq et al. [28], who reported a mean age of 44.7 years.

The study aimed to evaluate the diagnostic accuracy of multiparametric 3T MRI in the characterization of breast lesions, particularly malignant lesions, using BI-RADS criteria. Patients with BI-RADS categories 0, 1, and 6, isolated ductal ectasia, lack of consent, or absence of pathological follow-up were excluded.

The mean size of malignant breast lesions in this study was 3 cm, which is consistent with findings by Gheonea et al. [29] (2.7 cm), Yoon et al. [30] (2.9 cm), and Kim et al. [31] (2.8 cm). Clinically, all patients, 62 (100%), presented with a breast lump, followed by axillary swelling, 56 (90%), nipple discharge, 18 (30%), pain, 12 (19%), nipple retraction, nine (15%), skin change, six (10%), and fever, one (2%). Malignant lesions were more frequently associated with skin changes, whereas benign lesions were more commonly associated with pain, nipple discharge, and fever. Similar trends were observed in studies by Tawfeeq et al. [28] and Yoon et al. [30].

Morphologically, out of 62 participants in our study, the majority of lesions were oval in shape, 38 (61%), followed by irregular, 17 (28%), and round, seven (12%), aligning with findings by Uematsu et al. [32], who reported 59% oval, 26% irregular, and 15% round lesions. Most malignant lesions in the present study demonstrated heterogeneous enhancement, 39 (62.7%), while 23 (37.3%) showed homogeneous enhancement, consistent with the observations of Tan et al. [33].

Regarding lesion margins, irregular margins were most common, 34 (55.2%), followed by spiculated, 18 (29.8%), and lobulated, nine (15%), which is in agreement with previous studies [33]. NME lesions predominantly exhibited heterogeneous enhancement, 39 (62.7%), similar to prior reports.

Analysis of enhancement kinetics revealed that the majority of the lesions, 56 (89.5%), demonstrated type III (washout) curves, characterized by early enhancement followed by rapid washout, a pattern strongly associated with malignancy. This is consistent with findings by Schnall et al. [34], who reported that 76% of washout curves were associated with breast cancer. Type II (plateau) curves accounted for five (7.5%) of cases and are considered indeterminate, while type I (persistent) curves, typically associated with benign lesions, were observed in a small proportion. However, Schnall et al. [34] also reported persistent enhancement in 45% of malignant lesions, highlighting some variability compared to the current findings.

The diagnostic performance of multiparametric MRI in this study showed a sensitivity of 93%, a specificity of 60%, a PPV of 96.4%, and an NPV of 42.9%, with an AUC of 96.8%, indicating high diagnostic accuracy (p=0.008). These results are consistent with previous studies. Pinker et al. [24] demonstrated that multiparametric MRI using three parameters achieved a significantly higher AUC (0.936) compared to DCE-MRI alone (0.814). Similarly, Luo et al. [35] reported that combining multiple parameters improved diagnostic performance (AUC 0.965), with a high sensitivity (91%), specificity (95%), and overall accuracy (91.9%).

In addition to imaging characteristics, demographic and reproductive factors were also evaluated for their potential association with malignancy. Parity is an important epidemiological factor in breast cancer, as reproductive history influences hormonal exposure and breast tissue remodeling. In the present study, a higher proportion of malignant lesions was observed among multiparous women. However, this association did not reach statistical significance, likely due to the small sample size and the predominance of malignant cases in the study population. Larger population-based studies are required to better elucidate the relationship between parity and breast cancer risk.

Limitations

The primary limitation of this study is the relatively small sample size, which may be attributed to its single-center design and the inclusion of patients only from the Regional Institute of Medical Sciences Surgery Department. Additionally, the limited number of benign lesions may have influenced the overall analysis.

## Conclusions

Multiparametric 3T MRI is a highly accurate modality for differentiating benign and malignant breast lesions. The integration of DCE-MRI, DWI, and MR spectroscopy enhances lesion characterization by providing complementary morphological and functional information. Strong correlation with histopathological findings underscores its value in accurate diagnosis and clinical decision-making in patients with suspected breast malignancy.
